# The impact of team psychological safety, occupational coping efficacy, and job satisfaction on the job performance of operating room nurses: an empirical study based on structural equation modeling

**DOI:** 10.3389/fpubh.2026.1823932

**Published:** 2026-07-16

**Authors:** Lili Liu, Qiuyan Zhang, Lihua Yu, Bili Wang, Cui Chen, Ruiyu Li, Feng Cai

**Affiliations:** 1 Xiaolan Clinical Institute of Shantou University Medical College, Zhongshan, China; 2 Xiaolan People's Hospital of Zhongshan (The Fifth People's Hospital of Zhongshan), Zhongshan, China

**Keywords:** job performance, job satisfaction, occupational coping self-efficacy, operating room nurses, team psychological safety

## Abstract

**Background:**

The quality and efficiency of operating room nurses' work are directly related to patient safety, surgical success rates, and the overall medical quality of the hospital. In the context of a global shortage of nursing human resources, enhancing the job performance of operating room nurses is critically important. Team psychological safety, occupational coping efficacy, and job satisfaction are important factors affecting the job performance of operating room nurses. However, limited research has examined the interrelationships among these factors specifically within this specialized group. This study aims to explore the impact of team psychological safety on the job performance of operating room nurses, with a particular focus on the potential mediating roles of occupational coping efficacy and job satisfaction.

**Methods:**

A cross-sectional survey was conducted among operating room nurses from six general hospitals in southern China. A total of 308 questionnaires were distributed, and 303 valid responses were included in the final analysis (response rate: 98.38%). Data were collected using the Team Psychological Safety Climate Scale, Occupational Coping Self-Efficacy Scale, Job Satisfaction Scale, and Nurse Job Performance Scale. Descriptive statistics and Pearson correlation analyses were performed using SPSS 26.0, and structural equation modeling (SEM) was conducted using AMOS 25.0. Mediation effects were tested using the Bootstrap method.

**Results:**

Operating room nurses reported a high level of job performance (mean = 4.12, SD = 0.49). Team psychological safety was positively associated with occupational coping self-efficacy, job satisfaction, and job performance (*r* = 0.222–0.339, all *P* < 0.01). The structural equation model showed a good fit (χ^2^/df = 1.175, CFI = 0.989, RMSEA = 0.024). Team psychological safety significantly predicted job performance (β = 0.226, *P* < 0.01), with occupational coping self-efficacy and job satisfaction forming a significant serial mediation pathway (β = 0.247, *P* < 0.01).

**Conclusion:**

Team psychological safety significantly enhances the job performance of operating room nurses, both directly and indirectly through occupational coping self-efficacy and job satisfaction. These findings suggest that improving the psychological safety climate within teams may be an effective strategy to boost nursing performance in high-stakes clinical environments.

## 1 Introduction

Nursing human resource shortage has become an increasingly severe global issue (1, 2) with predictions of a global deficit of 4.5 million nurses by 2030 (3, 4). As a key component of healthcare systems, the nursing profession is facing unprecedented pressure, driven primarily by population aging, increased life expectancy, and the growing frequency of global public health emergencies (5, 6). China, as a middle-income country, confronts the unique challenge of “growing old before becoming rich”. Since entering an aging society in 2000, its older adults population has continued to grow (7), and the current situation of nursing human resources in China remains concerning (8, 9). Recent data indicate that China has only 3 nurses per 1,000 population (10), compared to an average of 9.2 per 1,000 in developed countries (11). This disparity underscores the urgent need to enhance nurse job performance.

The operating room (OR) is a highly specialized hospital department characterized by a complex, high-risk, and high-pressure environment that requires precise timing and strong collaboration. Given these characteristics, the quality and efficiency of OR nursing work are directly linked to patient safety, surgical success rates, and the overall standard of hospital care (12). OR play a vital role in the nursing team, assuming responsibilities such as preoperative preparation, intraoperative assistance, and postoperative management. Their job performance is therefore crucial to ensuring both patient outcomes and healthcare quality. Against the backdrop of ongoing nursing shortages and increasing job demands, identifying strategies to improve OR nurses' performance is of significant practical importance. Team psychological safety (13) is defined as a shared belief among team members that the team is safe for interpersonal risk-taking, such as speaking up, asking questions, or admitting mistakes. Although conceptualized as a team-level construct, it is commonly assessed through individual perceptions of the team climate. When individuals perceive a high level of psychological safety within their team, they are more likely to demonstrate proactive behaviors and engage constructively with their team (14, 15). However, the specific influence of team psychological safety on OR nurses' performance and the underlying mechanisms involved remain underexplored. Occupational coping self-efficacy, a concept introduced by Bandura, is defined as an individual's belief in their capacity to adapt to various professional situations and resolve work-related challenges. This is considered a core competency for nurses (16). Studies have shown that nurses with high occupational coping self-efficacy tend to exhibit better occupational mental health and are more likely to actively engage in their work (17, 18). Job satisfaction also plays a critical role in shaping nursing efficiency and the quality of care, directly influencing patient experience and healthcare safety. Low job satisfaction can contribute to staff turnover and instability within clinical teams (19). While prior studies have examined these variables independently, few have investigated how occupational coping efficacy and job satisfaction may mediate the relationship between team psychological safety and the job performance of OR nurses. Therefore, this study aims to explore the internal relationships and interaction mechanisms among team psychological safety, occupational coping self-efficacy, job satisfaction, and the job performance of OR nurses.

## 2 Background

### 2.1 Team psychological safety and job performance

Team psychological safety (TPS) refers to a shared belief among team members that they feel comfortable speaking up and taking interpersonal risks within the team, thereby facilitating the successful completion of team tasks (20, 21). This construct reflects a collective team climate, which is typically operationalized through individual-level perceptions aggregated to represent the team context. Previous research has shown that psychological safety not only reduces uncertainty during collaboration and fosters a sense of comfort in sharing information—thus enhancing team identification (2, 14, 22), but also encourages employees to engage in organizational voice behaviors, which in turn contributes to improved team performance (23). According to social information processing theory, the way individuals interpret and respond to environmental cues significantly shapes their attitudes and behaviors (24). In the workplace, TPS functions as a critical form of social information that directly influences employee performance (2, 23). It reflects team members' perceptions of their freedom and safety to express ideas, provide suggestions, and share perspectives within the team environment. When this perception is strong, employees are more likely to actively participate, share knowledge, and engage in innovative thinking, thereby enhancing both work efficiency and quality (15, 25). In contrast, low levels of psychological safety may contribute to employee silence and reduced collaboration, ultimately hindering team progress. However, existing research on TPS has predominantly focused on students and employees in business or organizational contexts, with limited exploration in high-stakes healthcare environments such as operating rooms. Based on the above considerations, we propose the following hypothesis:

*Hypothesis 1: Team psychological safety positively promotes the job performance of operating room nurses*.

### 2.2 The mediating role of occupational coping self-efficacy

#### 2.2.1 Self-efficacy

Occupational Coping Self-Efficacy (OCSE) refers to an individual's belief in their ability to effectively cope with challenges and pressures in the occupational environment. At its core, it reflects a person's positive appraisal of their own occupational competence and sense of control (18, 26). According to the Job Demands–Resources (JD-R) model (27), job resources play a crucial role in enhancing individuals' personal resources by reducing work-related strain and fostering adaptive coping mechanisms. In this context, TPS, as a key social and organizational resource, creates a supportive environment characterized by trust, open communication, and reduced interpersonal risk. Such an environment enables individuals to interpret job demands more positively and enhances their perceived control over work-related challenges (27, 28). Therefore, drawing on the resource activation mechanism of the JD-R model, TPS is expected to enhance OCSE, which in turn contributes to improved job performance. Previous studies have also suggested that TPS influences OCSE, which in turn mediates the impact on employee performance (29). Accordingly, this study proposes the following hypothesis:

*Hypothesis 2: Occupational coping self-efficacy mediates the relationship between team psychological safety and job performance*.

### 2.3 The mediating role of job satisfaction

Job satisfaction is a comprehensive evaluation by employees of their work environment, tasks, and rewards (30). It encompasses both extrinsic factors-such as compensation, promotion opportunities-and intrinsic experiences like autonomy and interpersonal relationships (31). Particularly in high-pressure, collaboration-intensive settings, job satisfaction serves as a critical predictor of employee stability, engagement, and performance (32, 33).

Within the framework of the JD-R model (27), job satisfaction can be conceptualized as an affective outcome resulting from the availability of job resources. When TPS provides a psychologically safe and supportive team environment, it reduces emotional exhaustion, enhances interpersonal trust, and strengthens employees' sense of belonging and value recognition, thereby promoting higher levels of job satisfaction (25, 34). Furthermore, according to the Conservation of Resources (COR) theory (23, 35, 36), individuals are motivated to acquire, maintain, and invest resources. In this context, job satisfaction represents a valuable psychological resource that can stimulate positive work attitudes and sustained engagement. Employees with higher job satisfaction are more likely to invest additional effort, maintain focus, and demonstrate proactive behaviors, which ultimately enhance job performance (36–39). Empirical evidence supports that employees with higher job satisfaction are more likely to demonstrate increased focus, greater problem-solving capacity, and stronger organizational citizenship behavior. These behaviors directly contribute to task performance and indirectly enhance team collaboration, thereby boosting overall work outcomes (40, 41). Therefore, integrating the JD-R and COR theoretical perspectives, job satisfaction is expected to function as an affective mediating mechanism linking TPS and job performance. Based on this theoretical and empirical rationale, the study proposes the following hypothesis:

*Hypothesis 3: Job satisfaction mediates the relationship between team psychological safety and job performance*.

### 2.4 The serial mediating role of occupational coping self-efficacy and job satisfaction

#### 2.4.1 Self-efficacy and job satisfaction

Team psychological safety, perceived by team members as group inclusiveness and support for interpersonal risk-taking (42, 43), plays a critical role in shaping both cognitive and emotional responses in high-pressure work environments such as operating rooms.

Integrating the JD-R model (27)and COR theory (23, 35, 36), the relationships among TPS, OCSE, job satisfaction, and job performance can be conceptualized as a sequential resource activation process. Specifically, TPS, as a contextual job resource, first facilitates the development of personal cognitive resources (i.e., OCSE) by reducing interpersonal risk, promoting open communication, and enhancing perceived control over work demands (25, 34). Subsequently, enhanced OCSE enables individuals to cope more effectively with job demands and to appraise work-related challenges more positively. This adaptive cognitive appraisal process contributes to more favorable emotional experiences, thereby fostering higher levels of job satisfaction as an affective resource (44). Finally, according to COR theory, accumulated psychological resources such as job satisfaction can motivate individuals to invest additional effort and maintain sustained work engagement, which ultimately translates into improved job performance through enhanced task execution and organizational citizenship behaviors (44, 45). This sequential pathway reflects a progressive mechanism of “job resource → personal cognitive resource → affective resource → behavioral outcome,” providing a coherent theoretical explanation for the serial mediating roles of OCSE and job satisfaction. Based on this integrated theoretical framework, the hypotheses are summarized as follows:

*H1: Team psychological safety positively predicts job performance among operating room nurses*.
*H2: Occupational coping self-efficacy mediates the relationship between team psychological safety and job performance*.
*H3: Job satisfaction mediates the relationship between team psychological safety and job performance*.
*H4: Occupational coping self-efficacy and job satisfaction sequentially mediate the relationship between team psychological safety and nurses' job performance*.

## 3 Methods

### 3.1 Design and participants

This study employed a cross-sectional design, in accordance with the STROBE (Strengthening the Reporting of Observational Studies in Epidemiology) guidelines (see Supplementary File 1). A convenience sampling method was used to recruit participants from six general hospitals in southern China. The inclusion criteria were as follows: 1) Possession of a valid nursing license and official registration; 2) Employment in an operating room for at least 1 year, with no severe physical, mental, or cognitive disorders, and the ability to engage in research-related activities; 3) Basic literacy and comprehension abilities, as well as proficiency in using communication tools such as WeChat; 4) Voluntary consent to participate in the study. The exclusion criteria were:1) Operating room nurses who withdrew from the study prior to completion; 2) Those unable to participate due to extraordinary circumstances during the study period.

### 3.2 Data collection

**Figure 1:**
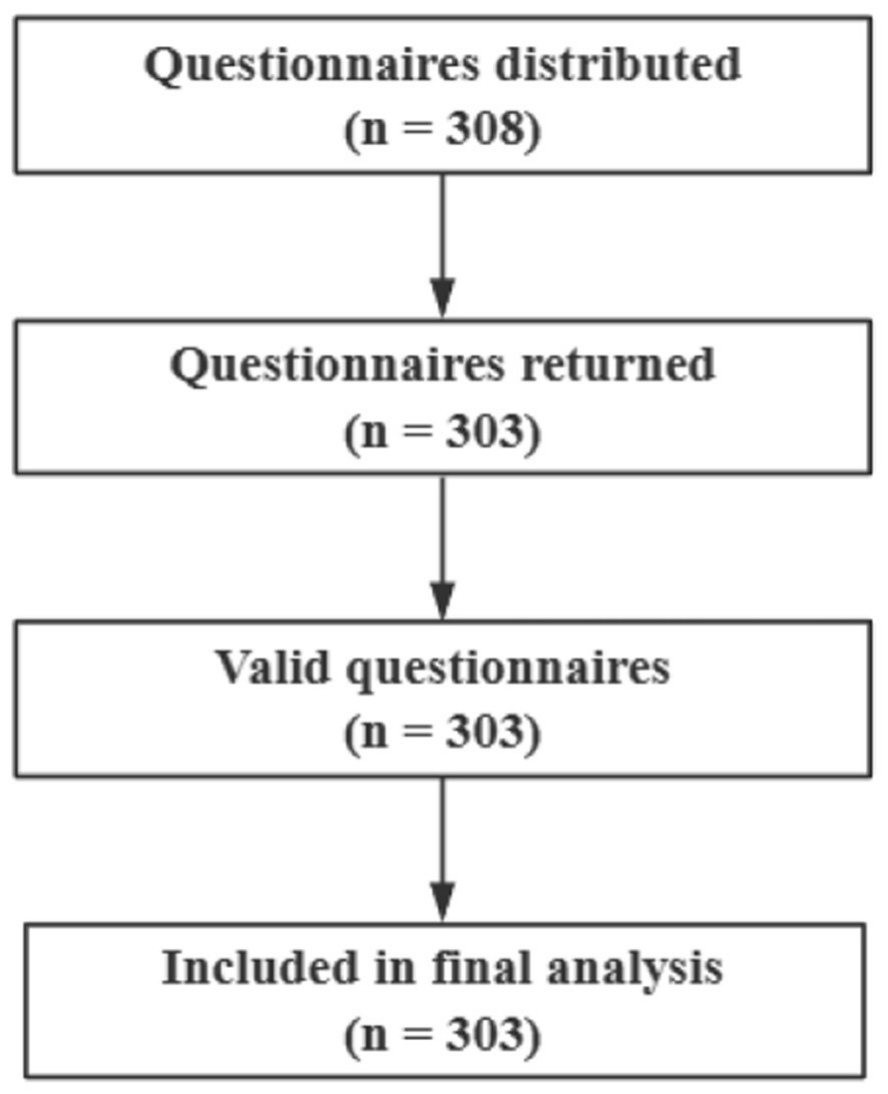
Participant recruitment and inclusion.

Under the supervision of well-trained research assistants, participants completed a structured questionnaire assessing demographic characteristics, team psychological safety, occupational coping self-efficacy, job satisfaction, and nursing job performance. Prior to data collection, the researchers contacted the head nurses of the operating rooms in each participating hospital to obtain their support and coordinate key details, including the survey schedule, locations, and estimated number of participants. During the survey, the researchers explained the study's purpose, procedures, and potential risks to all participants. Informed consent was obtained before the questionnaires were distributed. Data collection took place between March and April 2025. A total of 308 questionnaires were distributed, and 303 valid questionnaires were returned, yielding a response rate of 98.38%. These 303 responses were included in the final analysis. The participant recruitment and inclusion process is illustrated in Figure 1. Previous studies suggest that a sample size of ≥200 is considered adequate for structural equation modeling (SEM) analysis (46–48). Therefore, the sample size in this study satisfies the minimum requirement for SEM-based hypothesis testing.

### 3.3 Measurement

#### 3.3.1 Psychological safety climate team scale

This scale was developed by Edmondson (14) and includes four dimensions: speaking up, mutual respect, interpersonal risk-taking, and trust, with a total of 16 items., comprising a total of 16 items. Each item is rated on a 5-point Likert scale ranging from 1 (strongly disagree) to 5 (strongly agree), with total scores ranging from 16 to 80. Higher scores reflect a greater perception of psychological safety within the team. Although TPS is conceptually a team-level construct, it was measured in this study at the individual level as nurses' perceived team psychological safety, and each participant's response was treated as an independent observation. Given the limited number of clusters, multilevel modeling was not conducted, which is consistent with prior research using individual-level perceptions of team climate. The scale has demonstrated good reliability and validity in the Chinese cultural context, with a reported Cronbach's α of 0.832 (49). Confirmatory factor analysis (CFA) was used to test the compatibility of the scale structure with the collected data, and the results showed an acceptable fit between the scale's factor structure and the data (χ^2^/df = 2.53, GFI = 0.91, AGFI=0.90, CFI = 0.94, IFI=0.92, RMSEA = 0.04, SRMR = 0.03). In addition, the reliability (Cronbach's alpha) of this scale in this study was 0.90.

#### 3.3.2 Occupational coping self-efficacy scale

The Occupational Coping Self-Efficacy Scale was developed by Pisant (50), and is used to assess nurses' confidence in managing occupational demands. The scale consists of two dimensions: occupational burden (6 items) and interpersonal relationship difficulties (3 items), totaling 9 items. Each item is scored on a 5-point Likert scale ranging from 1 (none) to 5 (a great deal), yielding a total score between 9 and 45. Higher scores indicate greater perceived coping efficacy. This instrument has shown good psychometric properties in the Chinese context, with a previously reported Cronbach's α of 0.882 (51). CFA results revealed a satisfactory construct validity (χ^2^/df = 2.57, GFI = 0.97, AGFI=0.93, CFI = 0.92, IFI=0.90, RMSEA = 0.06, SRMR = 0.05). In addition, the reliability (Cronbach's alpha) of the scale in this study was found to be 0.87.

#### 3.3.3 Job satisfaction scale

The Job Satisfaction Scale was developed by Weiss et al. (52) and includes three dimensions: intrinsic satisfaction (12 items), extrinsic satisfaction (6 items), and general satisfaction (2 items), totaling 20 items. Each item is rated on a 5-point Likert scale, with total scores ranging from 20 to 100. Higher scores reflect a more positive overall evaluation of one's job. The scale has demonstrated good reliability and validity in the Chinese cultural context (Cronbach's α = 0.84) (53). The CFA results indicated a good model fit (χ^2^/df = 315, GFI = 0.95, AGFI=0.93, CFI = 0.92, IFI=0.90, RMSEA = 0.06, SRMR = 0.04). In addition, the reliability (Cronbach's alpha) of the scale in this study was found to be 0.93.

#### 3.3.4 Nurse job performance scale

The Nurse Job Performance Scale was developed by Jansson-Frojmark et al. (54)), is an effective tool for assessing nursing performance. It comprises three dimensions: team collaboration, work proactivity, and work engagement, with a total of 14 items. Each item is rated on a 5-point Likert scale, ranging from 1 (completely disagree) to 5 (completely agree), resulting in a total score ranging from 14 to 70. Higher scores indicate higher levels of nursing job performance. The scale has demonstrated good psychometric properties in the Chinese context, with a reported Cronbach's α of 0.853 (55). The CFA results indicated a good model fit (χ^2^/df = 2.86, GFI = 0.94, AGFI=0.93, CFI = 0.90, IFI=0.96, RMSEA = 0.05, SRMR = 0.06). In addition, the reliability (Cronbach's alpha) of the scale in this study was found to be 0.89.

### 3.4 Data analysis

Data analysis and model construction were conducted using SPSS 26.0 and AMOS 25.0. Descriptive statistics were first used to analyze the demographic characteristics of participants. Given that all variables were measured using self-report instruments, the Harman single-factor test was employed to assess common method bias (CMB). The normality of continuous variables was assessed using the Kolmogorov–Smirnov test, with a *P* > 0.05 indicating a normal distribution (56). For variables that followed a normal distribution, Pearson correlation analysis was conducted to examine the relationships among the four core variables and their dimensions. For variables that did not meet the assumption of normality, Spearman's rank correlation analysis was applied. Correlation coefficients were interpreted as follows: negligible (0 < *r* < 0.2), weak (0.2 ≤ *r* < 0.4), moderate (0.4 ≤ *r* < 0.6), and strong (*r* ≥ 0.6 (57). Subsequently, a structural equation model (SEM) was employed to test the mediating roles of occupational coping self-efficacy and job satisfaction in the relationship between team psychological safety and nurse job performance (58). Model fit was evaluated using the following indices: χ^2^/df, GFI, AGFI, CFI, IFI, RMSEA, and SRMR. The model was considered to have acceptable fit if χ^2^/df < 5, GFI > 0.90, AGFI > 0.90, CFI > 0.90, IFI > 0.90, SRMR < 0.05, and RMSEA ≤ 0.08 (58). To test the significance of the mediating effects, the bias-corrected percentile Bootstrap method was applied with 5,000 resamples, estimating the 95% confidence interval (CI) for indirect effects. Mediation was considered statistically significant if the 95% CI did not include zero (59).

### 3.5 Ethical considerations

This study was reviewed and approved by the Ethics Committee of Xiaolan People's Hospital, Zhongshan City (No. ZSXL-LL2025-043), and was conducted in accordance with the Declaration of Helsinki. All participants were informed of the study purpose and procedures, and written informed consent was obtained prior to data collection. Participation was voluntary, and confidentiality and anonymity were assured throughout the study.

## 4 Results

### 4.1 Common method bias test

Given that all variables were assessed using self-reported measures, the potential for common method bias was evaluated. Harman's single-factor test identified 16 factors with eigenvalues greater than 1, with the first factor accounting for 23.71% of the total variance. As this value is below the commonly accepted threshold of 40%, common method bias was not considered a serious concern in this study.

### 4.2 Participant characteristics

A total of 303 operating room nurses were included, comprising 52 males (17.2%) and 251 females (82.8%). Nearly half were aged over 35 years (48.8%), and most were married (77.2%). The majority held a bachelor's degree (79.9%). Most participants had over 10 years of work experience (65.7%), and 67.0% held intermediate or senior professional titles. Additionally, 46.5% had two or more children, 46.9% reported a monthly income of 6,000–10,000 yuan, 55.8% were permanently employed, and 49.5% worked four or more night shifts per month. Detailed demographic characteristics are shown in Table 1.

**Table 1:** Demographic characteristics of the participants (*N* = 303).

Variables		*N*	%
Gender	Male	52	17.2%
	Female	251	82.8%
Age	Less than 25 years old	21	6.9%
	25–30 years old	58	19.1%
	31–35 years old	76	25.1%
	More than 35 years old	148	48.8%
Marital status	Single	62	20.5%
	Married	234	77.2%
	Divorced or Widowed	7	2.3%
Educational attainment	Junior college and below	54	17.8%
	Bachelor's degree	242	79.9%
	Master's degree or above	7	2.3%
Years of work experience	Less than 5 years	42	13.9%
	5–10 years	62	20.5%
	More than 10 years	199	65.7%
Professional title	Junior title	100	33.0%
	Intermediate title	148	48.8%
	Senior title	55	18.2%
Number of children	No children	76	25.1%
	One child	86	28.4%
	Two or more children	141	46.5%
Monthly income	Less than 6,000 yuan	52	17.2%
	6,000–10,000 yuan	142	46.9%
	More than 10,000 yuan	109	36.0%
Employment type	In–service	169	55.8%
	Non–in–service	134	44.2%
Average monthly night shifts	Less than 4 times	153	50.5%
	4–6 times	114	37.6%
	More than 6 times	36	11.9%

### 4.3 Descriptive statistics and correlation analysis

The Kolmogorov–Smirnov test results indicated that the variables-team psychological safety, occupational coping self-efficacy, job satisfaction, and nursing job performance-were all normally distributed (*P* > 0.05). Accordingly, Pearson correlation analysis was employed to examine the relationships among the four variables. The results are summarized in Table 2. The mean scores (± standard deviations) were 3.37 ± 0.50 for team psychological safety, 3.70 ± 0.65 for occupational coping self-efficacy, 3.64 ± 0.68 for job satisfaction, and 4.12 ± 0.49 for nursing job performance. The results revealed that team psychological safety was positively correlated with occupational coping self-efficacy (*r* = 0.222, *P* < 0.01), job satisfaction (*r* = 0.294, *P* < 0.01), and nursing job performance (*r* = 0.339, *P* < 0.01). Occupational coping self-efficacy was positively correlated with job satisfaction (*r* = 0.314, *P* < 0.01) and nursing job performance (*r* = 0.362, *P* < 0.01), while job satisfaction was positively correlated with nursing job performance (*r* = 0.446, *P* < 0.01). These findings support the hypothesized positive associations among the study variables and provide a basis for the subsequent mediation analysis.

**Table 2:** Descriptive statistics and correlation analysis (*r*).

variables	DE	MR	IR	RT	TPS	OB	ID	OCSE	IS_	ES	GS	JS	WC	WE	WI	NWP	Score Range	Mean ±Error
**DE**	1																**1–5**	3.38 ± 0.72
**MR**	0.485^**^	1															**1–5**	3.30 ± 0.65
**IR**	0.395^**^	0.404^**^	1														**1–5**	3.47 ± 0.73
**RT**	0.497^**^	0.379^**^	0.319^**^	1													**1–5**	3.34 ± 0.59
**TPS**	0.802^**^	0.754^**^	0.728^**^	0.706^**^	1												**1–5**	3.37 ± 0.50
**OB**	0.187^**^	0.178^**^	0.166^**^	0.156^**^	0.230^**^	1											**1–5**	3.72 ± 0.73
**ID**	0.075	0.116^*^	0.106	0.072	0.124^*^	0.448^**^	1										**1–5**	3.67 ± 0.82
**OCSE**	0.171^**^	0.180^**^	0.168^**^	0.145^*^	0.222^**^	0.928^**^	0.749^**^	1									**1–5**	3.70 ± 0.65
**IS_**	0.275^**^	0.162^**^	0.226^**^	0.231^**^	0.300^**^	0.276^**^	0.212^**^	0.293^**^	1								**1–5**	3.54 ± 0.85
**ES**	0.162^**^	0.119^*^	0.100	0.151^**^	0.176^**^	0.246^**^	0.171^**^	0.254^**^	0.443^**^	1							**1–5**	30.80 ± 00.73
**GS**	0.087	0.115^*^	0.059	00.107	0.121^*^	0.129^*^	00.081	0.129^*^	0.483^**^	0.435^**^	1						**1–5**	3.81 ± 0.85
**JS**	0.266^**^	0.172^**^	0.207^**^	0.233^**^	0.294^**^	0.299^**^	0.222^**^	0.314^**^	0.943^**^	0.702^**^	0.620^**^	1					**1–5**	3.64 ± 0.68
**WC**	0.176^**^	0.184^**^	0.192^**^	00.106	0.224^**^	0.267^**^	0.238^**^	0.297^**^	0.300^**^	0.305^**^	0.156^**^	0.339^**^	1				**1–5**	4.16 ± 0.61
**WE**	0.170^**^	0.212^**^	0.220^**^	0.238^**^	0.278^**^	0.247^**^	0.172^**^	0.255^**^	0.358^**^	0.245^**^	0.197^**^	0.368^**^	0.348^**^	1			**1–5**	40.14 ± 00.63
**WI**	0.218^**^	0.222^**^	0.276^**^	0.185^**^	0.304^**^	0.258^**^	0.225^**^	0.285^**^	0.298^**^	0.261^**^	0.249^**^	0.335^**^	0.446^**^	0.390^**^	1		**1–5**	40.05 ± 00.67
**NWP**	0.241^**^	0.262^**^	0.290^**^	0.216^**^	0.339^**^	0.333^**^	0.277^**^	0.362^**^	0.407^**^	0.354^**^	0.252^**^	0.446^**^	0.833^**^	0.703^**^	0.770^**^	1	**1–5**	4.12 ± 0.49

^**^. At the 0.01 level (two–tailed), the correlation is significant.

TPS, Team Psychological Safety; DE Direct Expression, MR Mutual Respect, IR Interpersonal Risk-Taking, RT Reciprocal Trust; OCSE, Occupational Coping Self-Efficacy; OB, Occupational Burden; ID, Interpersonal Difficulty; JS, Job Satisfaction; IS Internal Satisfaction; ES, External Satisfaction; GS, General Satisfaction; NWP, Nurse Work Performance; WC, Team Collaboration; WE, Work Enthusiasm; WI, Work Engagement.

### 4.4 Mediation effect results and fit indices

**Figure 2:**
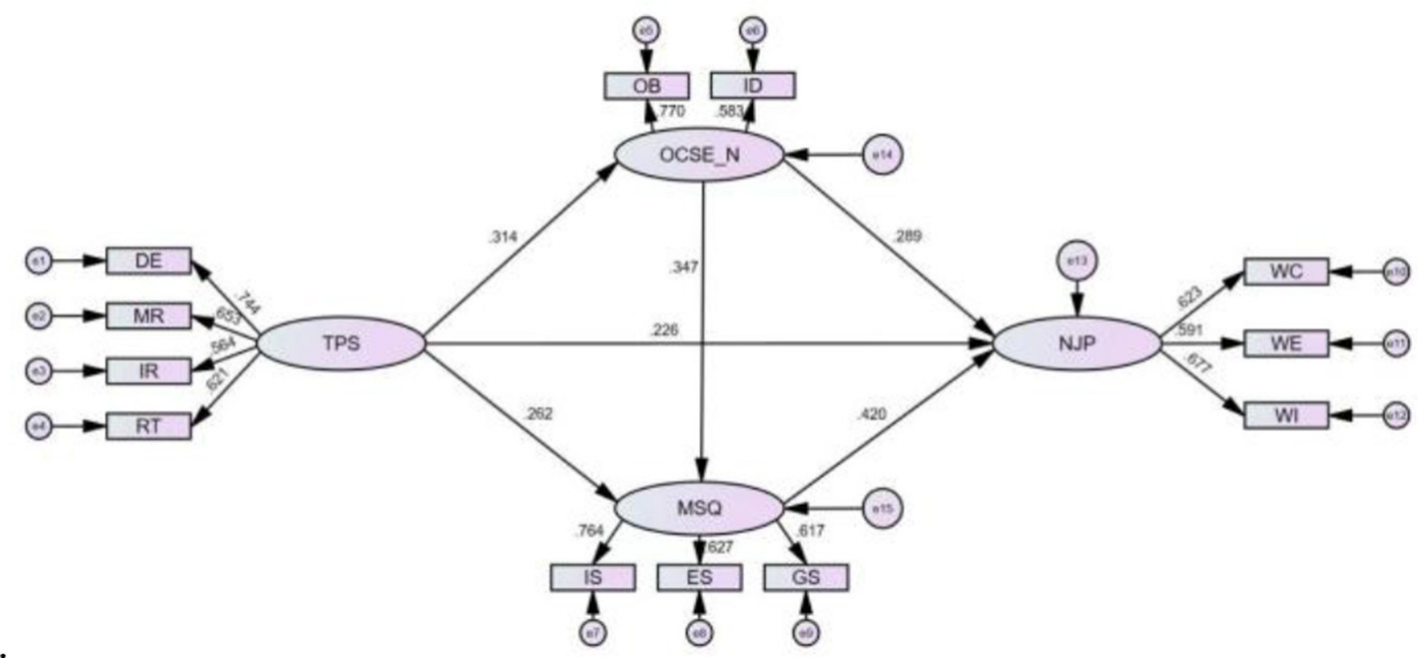
The mediating role of occupational coping self-efficacy and job satisfaction between team psychological safety and nurse job performance. Abbreviation: DE, MR, IR, RT: Four dimensions of team psychological safety; OB, ID: Two dimensions of occupational coping self-efficacy; IS, ES, GS: Three dimensions of job satisfaction; WC, WE, WI: Three dimensions of nurse Job performance.

The results demonstrated that team psychological safety positively predicted occupational coping self-efficacy and job satisfaction (β = 0.314, *P* < 0.01; β = 0.262, *P* < 0.01), while occupational coping self-efficacy positively predicted job satisfaction (β = 0.347, *P* < 0.01). Furthermore, team psychological safety, occupational coping self-efficacy, and job satisfaction each had significant positive predictive effects on nursing job performance (β = 0.226, *P* < 0.01; β = 0.289, *P* < 0.01; β = 0.420, *P* < 0.01), thereby supporting Hypothesis 1. To examine the mediating effects, the percentile bootstrap and bias-corrected bootstrap methods were applied with 5,000 resamples to calculate the 95% CIs. The results indicated that both occupational coping self-efficacy and job satisfaction served as mediators in the relationship between team psychological safety and nursing job performance, with a total indirect effect of 0.247. Specifically, the indirect effect through occupational coping self-efficacy alone was 0.091 (95% CI: 0.015–0.227, *P* < 0.01), supporting Hypothesis 2. The indirect effect via job satisfaction alone was 0.110 (95% CI: 0.027–0.252, *P* < 0.01), supporting Hypothesis 3. Additionally, the serial mediation pathway—team psychological safety → occupational coping self-efficacy → job satisfaction → nursing job performance—yielded an indirect effect of 0.046 (95% CI: 0.013–0.120, *P* < 0.01), supporting Hypothesis 4 (see Figure 2 and Table 3). The structural model demonstrated a good fit to the data, as indicated by the following fit indices: χ^2^/df = 1.175, GFI = 0.971, AGFI = 0.953, NFI = 0.932, RFI = 0.907, IFI = 0.989, TLI = 0.985, CFI = 0.989, RMR = 0.020, and RMSEA = 0.024, suggesting that the hypothesized model was acceptable (see Table 4).

**Table 3:** Standardized bootstrap mediation effect test.

Effect	Path	Standardized *β*	SE	The size of effect	95%CI			Hypothesis
					Lower	Upper	*P*	
Total	TPS → NJP	0.473						
Direct	TPS → NJP	0.226						Hypothesis1:support
Indirect	TPS → OCSE → NJP	0.091	0.053	19.24%	0.015	0.227	0.008	Hypothesis2:support
	TPS → JS → NJP	0.110	0.054	23.26%	0.027	0.252	0.005	Hypothesis3:support
	TPS → OCSE → JS → NJP	0.046	0.024	9.73%	0.013	0.120	0.002	Hypothesis4:support

TPS, Team psychological safety; OCSE, Occupational coping self–efficacy; JS, Job satisfaction; NWP, Nurse job performance.

**Table 4:** Model fit summary.

Model fit	X^2^/df	GFI	AGFI	NFI	RFI	IFI	TLI	CFI	RMR	RMSEA
Model–fitting standard	< 5	>0.90	>0.90	>0.90	>0.90	>0.90	>0.90	>0.90	< 0.05	≤ 0.08
Model–fitting index	1.175	0.971	0.953	0.932	0.907	0.989	0.985	0.989	0.020	0.024

## 5 Discussion

This study examined the relationships among team psychological safety, occupational coping self-efficacy, job satisfaction, and job performance among operating room nurses. The findings indicate that team psychological safety significantly enhances job performance. Moreover, occupational coping self-efficacy and job satisfaction both mediate this relationship, with a significant sequential mediation pathway (TPS → OCSE → JS → performance). Specifically, team psychological safety strengthens coping self-efficacy, which in turn increases job satisfaction and ultimately improves job performance. These results highlight the importance of fostering a psychologically safe team environment to enhance nurses' coping capacity, satisfaction, and overall performance in high-pressure clinical settings.

### 5.1 Team psychological safety and nurses' job performance

The results of this study demonstrate that team psychological safety significantly and positively predicts the job performance of operating room nurses, aligning with the findings of Ashley M. et al. (60) and Gabriela Fernández Castillo et al. (61). Given the high-pressure and task-intensive environment of operating rooms, fostering team psychological safety is particularly essential for this nursing subgroup. A psychologically safe team climate-characterized by openness, mutual trust, and support—can effectively alleviate psychological stress, bolster confidence, and enhance collaboration among nurses, ultimately contributing to improved job performance, job satisfaction, and organizational identification (15). By cultivating a supportive and transparent work environment, offering both emotional and material support, and establishing a culture of positive communication and mutual respect, operating room nurses are more likely to feel psychologically secure. This sense of safety, in turn, promotes proactive behavior, increases work engagement, and improves task efficiency, thereby ensuring the quality of intraoperative care and patient safety. In essence, team psychological safety serves as a foundational factor for improving performance and cohesion within surgical nursing teams, with downstream effects on care quality and institutional safety (25).

To strengthen psychological safety in the workplace, medical institutions should actively support the development of relevant competencies among operating room nurse managers. Nursing leadership should adopt people-centered and flexible management strategies, enhance autonomy, and ensure fair treatment in promotion and recognition. Specifically, nurse managers can implement structured communication practices (e.g., preoperative briefings and postoperative debriefings) to promote open dialogue, and establish non-punitive feedback systems (e.g., anonymous reporting and team reflection sessions) to encourage speaking up. In addition, targeted training programs focusing on stress management and simulation-based coping skills can be used to enhance occupational coping self-efficacy. Furthermore, addressing nurses' professional and personal needs through ongoing training, clear career pathways, and flexible scheduling can improve job satisfaction and organizational commitment. Mentorship and peer-support programs may further reinforce both psychological safety and coping capacity. These strategies can ultimately improve performance, team stability, and patient safety.

### 5.2 The mediating role of occupational coping efficacy

#### 5.2.1 Efficacy

In the healthcare setting, particularly within operating rooms, the occupational coping self-efficacy of nurses plays a pivotal role in determining their task execution effectiveness. This study confirms that team psychological safety significantly contributes to enhancing nurses' coping efficacy, consistent with prior research (30). Operating room nurses are routinely exposed to high-stress conditions and high-stakes procedures. Under such circumstances, they may experience self-doubt regarding their clinical competence, professional value, and contribution to the surgical team (12). This internalized uncertainty can lead to hesitation, increased anxiety, and impaired performance during complex tasks. However, when nurses perceive a high level of team psychological safety—manifested through mutual trust, respect, and collaborative support—they are more likely to confront work challenges with confidence and resilience (13). A psychologically safe environment empowers nurses to express ideas and concerns without fear of criticism, thereby strengthening their coping capacity. Nursing managers should take proactive measures to cultivate such an environment by organizing regular team-building activities, promoting open and transparent communication, and adopting a supportive leadership style. These strategies not only enhance interpersonal trust but also foster a culture of mutual respect and psychological security, thereby facilitating the development of occupational coping efficacy. Nurses with high occupational coping efficacy demonstrate greater adaptability in dynamic and high-pressure environments, enhanced ability to manage intraoperative emergencies, and improved overall work quality and efficiency. Therefore, nursing managers play an instrumental role in reinforcing this capacity. By providing constructive feedback, professional guidance, and timely support in addressing work-related challenges, they help nurses build resilience and confidence. Effective leadership and communication can transform difficult situations into opportunities for learning and growth, further reinforcing nurses' efficacy beliefs and performance.

From a practical perspective, nurse managers should implement targeted strategies to enhance occupational coping self-efficacy. In operating room settings characterized by high-stakes tasks, time pressure, and hierarchical structures, nurses must respond quickly and accurately to complex situations. Leadership is therefore critical; timely feedback, individualized guidance, and reflective learning can help nurses build confidence and competence under pressure. Healthcare institutions should adopt multi-level interventions, including peer-support and mentorship programs, targeted training, and appropriate workload allocation. Simulation-based training for intraoperative emergencies may be particularly effective in this context. These strategies can strengthen coping capacity, improve job performance, and support high-quality surgical care.

### 5.3 The mediating role of job satisfaction

Job satisfaction, as a mediating variable, is positively associated with job performance, aligning with the findings of Hatice Serap Koçak et al. (62). As a key element of organizational climate, team psychological safety fosters an environment where the voices of operating room nurses from diverse backgrounds are actively heard, respected, and valued. This openness and inclusiveness significantly influence nurses' work attitudes and behaviors (34). When nurses perceive a psychologically safe team environment, they are more likely to engage in positive, proactive communication with colleagues, interdepartmental staff, and patients and their families. This, in turn, cultivates stronger team cohesion and more effective interpersonal interactions. Such a collaborative and trusting work climate enhances nurses' job satisfaction and reinforces mutual trust between nurses and their colleagues and supervisors. Increased job satisfaction not only improves morale but also encourages a more committed and responsible approach to complex clinical tasks, thereby positively influencing overall job performance (25). In the Chinese healthcare context, where cultural norms and work environments differ from those in Western settings, the influence of team psychological safety on job satisfaction and performance is especially pronounced. Hierarchical relationships and high work demands often shape nurses' perceptions of their roles, making psychological support from leadership even more critical.

In this regard, nurse managers play a key role in shaping job satisfaction. In operating room settings characterized by high pressure and hierarchical structures, leadership behaviors and communication styles are particularly influential in nurses' emotional experiences and engagement. By adopting a supportive and empathetic approach, maintaining effective communication, and fostering strong nurse–manager relationships, leaders can enhance job satisfaction. This, in turn, promotes work engagement, improves team performance, and supports a stable and efficient operating room nursing team.

### 5.4 Chain mediation effects of occupational coping efficacy and job satisfaction

#### 5.4.1 Efficacy and job satisfaction

Beyond the direct effects observed, this study further identified a significant chain mediating effect of occupational coping efficacy and job satisfaction in the relationship between team psychological safety and nurses' work performance. Specifically, operating room nurses with high levels of occupational coping efficacy tend to exhibit greater confidence and competence in managing workplace challenges, which, in turn, enhances their job satisfaction (63**?** ). This heightened satisfaction fosters a more proactive and engaged attitude toward work, encouraging participation in team activities, fostering collaborative relationships, and strengthening overall team efficacy, sense of belonging, and job performance (25).

These findings highlight the need for healthcare institutions and nurse managers to leverage the sequential pathway linking psychological safety, coping efficacy, and job satisfaction. In operating room settings characterized by high-risk tasks, time pressure, and hierarchical structures, targeted interventions are particularly critical. Practical strategies include optimizing performance management systems to enhance fairness and transparency, thereby strengthening coping efficacy and job satisfaction. In addition, fostering a psychologically safe team environment—through open communication, mutual support, and trust—is essential. These approaches can improve team cohesion, enhance nurse engagement and performance, and ultimately promote safer and more effective patient care in the operating room.

## 6 Limitations of the study and future research

This study has several limitations. First, the use of convenience sampling, with participants drawn from six general hospitals in southern China, may limit the generalizability of the findings to broader populations or different healthcare settings. Second, the cross-sectional design employed in this study precludes any inference of causal relationships among the variables. Third, all variables were measured using self-report questionnaires collected at a single time point, which may introduce common method bias. Although Harman's single-factor test suggested that this bias was not a serious concern, its potential influence cannot be entirely ruled out. Fourth, although team psychological safety is conceptually a team-level construct, it was measured and analyzed at the individual level as perceived team psychological safety in this study. Due to the limited number of clusters, aggregation analyses (e.g., ICC or Rwg) and multilevel modeling were not conducted. This may introduce potential bias related to level-of-analysis mismatch and limit the interpretation of findings at the team level.

To address these limitations, future research should consider expanding the geographic scope and sample diversity by including hospitals from various regions and healthcare systems, thereby enhancing the external validity of the results. Additionally, longitudinal study designs are recommended to better capture dynamic changes and to examine the causal pathways linking team psychological safety, occupational coping efficacy, job satisfaction, and job performance over time. Future studies are also encouraged to adopt multi-source data or time-lagged designs to further reduce the risk of common method bias. Moreover, future research could employ multilevel designs and collect data from intact teams to validate team-level constructs using aggregation statistics and further strengthen the robustness of conclusions.

## 7 Conclusion

Operating room nurses play a vital role in surgical procedures and the care of critically ill patients. The quality of their work directly impacts surgical outcomes and patient safety; thus, enhancing their job performance is of critical importance. This study systematically examined the mechanisms through which team psychological safety influences the job performance of operating room nurses. The findings revealed a significant positive association between team psychological safety and job performance. Moreover, occupational coping self-efficacy and job satisfaction were identified as sequential mediators in this relationship. These findings provide a theoretical foundation for healthcare institutions to implement targeted strategies aimed at improving nurses' work performance. Specifically, the results underscore the importance of fostering a psychologically safe team environment, enhancing nurses' coping capabilities, and improving job satisfaction. Together, these approaches can contribute to better team collaboration, higher-quality nursing care, and improved patient outcomes in surgical settings.

## Data Availability

The original contributions presented in the study are included in the article/Supplementary material, further inquiries can be directed to the corresponding author.
